# Salicylic Acid against Tænia

**Published:** 1879-08

**Authors:** 


### SALICYLIC ACID AGAINST TÆNIA.

After trying almost all other remedies in vain, Maryrowski administered to a lady who had suffered with Tænia Solium for nine years, 8 grains of salicylic acid four times, at intervals of one hour, and then gave a tablespoonful of castor oil. The treatment proved painless and perfectly successful.

